# A novel computer-assisted tool for 3D imaging of programmed death-ligand 1 expression in immunofluorescence-stained and optically cleared breast cancer specimens

**DOI:** 10.1186/s12885-023-11748-8

**Published:** 2024-01-24

**Authors:** Yi-Hsuan Lee, Chung-Yen Huang, Yu-Han Hsieh, Chia-Hung Yang, Yu-Ling Hung, Yung-An Chen, Yu-Chieh Lin, Ching-Hung Lin, Jih-Hsiang Lee, Ming-Yang Wang, Wen-Hung Kuo, Yen-Yin Lin, Yen-Shen Lu

**Affiliations:** 1 Department of Pathology, National Taiwan University Hospital, Taipei, Taiwan; 2 JelloX Biotech Inc, Hsinchu, Taiwan; 3 Department of Medical Oncology, Cancer Center Branch, National Taiwan University Hospital, Taipei, Taiwan; 4 Department of Internal Medicine, National Taiwan University College of Medicine, Taipei, Taiwan; 5 Department of Oncology, National Taiwan University Hospital, Hsin-Chu Branch, Hsinchu, Taiwan; 6 Department of Surgical Oncology, Cancer Center Branch, National Taiwan University Hospital, Taipei, Taiwan; 7 Department of Surgery, National Taiwan University Hospital, Taipei, Taiwan; 8 Department of Oncology, National Taiwan University Hospital, No.7, Chung Shan S. Rd., Zhongzheng Dist, Taipei, 100225 Taiwan

**Keywords:** Breast cancer, Immunofluorescence staining, Optical clearing, Programmed death-ligand 1, Three-dimensional imaging

## Abstract

**Background:**

Programmed death-1 (PD-1) and programmed death-ligand 1 (PD-L1) are the two most common immune checkpoints targeted in triple-negative breast cancer (BC). Refining patient selection for immunotherapy is non-trivial and finding an appropriate digital pathology framework for spatial analysis of theranostic biomarkers for PD-1/PD-L1 inhibitors remains an unmet clinical need.

**Methods:**

We describe a novel computer-assisted tool for three-dimensional (3D) imaging of PD-L1 expression in immunofluorescence-stained and optically cleared BC specimens (n = 20). The proposed 3D framework appeared to be feasible and showed a high overall agreement with traditional, clinical-grade two-dimensional (2D) staining techniques. Additionally, the results obtained for automated immune cell detection and analysis of PD-L1 expression were satisfactory.

**Results:**

The spatial distribution of PD-L1 expression was heterogeneous across various BC tissue layers in the 3D space. Notably, there were six cases (30%) wherein PD-L1 expression levels along different layers crossed the 1% threshold for admitting patients to PD-1/PD-L1 inhibitors. The average PD-L1 expression in 3D space was different from that of traditional immunohistochemistry (IHC) in eight cases (40%). Pending further standardization and optimization, we expect that our technology will become a valuable addition for assessing PD-L1 expression in patients with BC.

**Conclusion:**

Via a single round of immunofluorescence imaging, our approach may provide a considerable improvement in patient stratification for cancer immunotherapy as compared with standard techniques.

**Supplementary Information:**

The online version contains supplementary material available at 10.1186/s12885-023-11748-8.

## Introduction

The global burden of breast cancer (BC) is substantial, with approximately 2.3 million new cases and 685,000 deaths worldwide in 2020 [1]. BC is a heterogeneous malignancy that is traditionally classified by the expression of specific hormone receptors (i.e., estrogen receptor [ER] and progesterone receptor [PR]), as well as the overexpression of human epidermal growth factor receptor 2 (HER2) [2–4]. With limited therapeutic options, triple-negative (i.e., negative for PR, ER, and HER2 receptors) BC appears to be associated with the least favorable outcomes among the major subtypes [4]. Under these circumstances, targeted therapy in search for non-endocrine targets is gaining attention [5, 6].

By addressing immune evasion through the activation of T cell-mediated cytotoxic responses, the development of immune checkpoint inhibitors (ICIs) has been a major breakthrough in the field of cancer immunotherapy [7–9]. PD-1 and PD-L1 are the two most common immune checkpoints targeted in BC [10]. Among the monoclonal antibodies that bind and block the PD-1/PD-L1 axis, atezolizumab (PD-L1-binding) and pembrolizumab (PD-1-binding) have received approval for patients with unresectable locally advanced or metastatic triple-negative BC expressing PD-L1 [7, 8]. Currently, eligibility to treatment with anti-PD-1/PD-L1 antibodies relies on the IHC detection of PD-L1 in tumor specimens [11–14]. However, heterogeneity of PD-L1 expression still poses significant technical challenges. The resulting risk of inaccurate or incorrect patient allocation to ICIs [15] calls for an optimized PD-L1 detection technique.

Optical clearing has emerged as an increasingly viable option to bypass the optical heterogeneity of tissue components and meet the growing demand for 3D tissue imaging [16]. Recent advances in 3D tissue clearing techniques [17, 18] – coupled with high-throughput computational analysis of optically cleared samples – have provided significant opportunities to explore tumor heterogeneity and its clinical significance [19–23]. While most previous 3D studies in the field have focused on visualizing tumor morphology in serial sections, there have been limited attempts to track single protein expression within the highly complex tumor microenvironment. In this proof-of-concept study, we describe a novel computer-assisted tool for 3D imaging of PD-L1 expression in immunofluorescence-stained and optically cleared BC specimens. The devised algorithm has the potential to improve current stratification schemes for allocating triple-negative BC patients to ICIs.

## Methods

### Patient cohort

All procedures were in accordance with the ethical standards established by the Declaration of Helsinki and the study protocol was approved by National Taiwan University Hospital (IRB reference number: 202004032RSB). Each participant provided written informed consent. Surgical or biopsy primary tumor specimens (n = 33) were prospectively collected from 33 adult women (> 18-years old) who had been diagnosed with BC (including six with triple-negative BC) at National Taiwan University Hospital between 2020 and 2022. Patients who had previously undergone treatment before specimen collection were excluded, as were pregnant or lactating women.

### Specimen sets

Primary tumor specimens (n = 33) were randomly divided into three distinct sets as follows: (1) samples used to perform autofluorescence testing (n = 3), (2) samples used to train computer-assisted tumor-infiltrating immune cell detection algorithm (n = 10), and (3) samples used to analyze PD-L1 expression using the computer-assisted algorithm (n = 20).

### Sample preparation

**Fig. 1:**
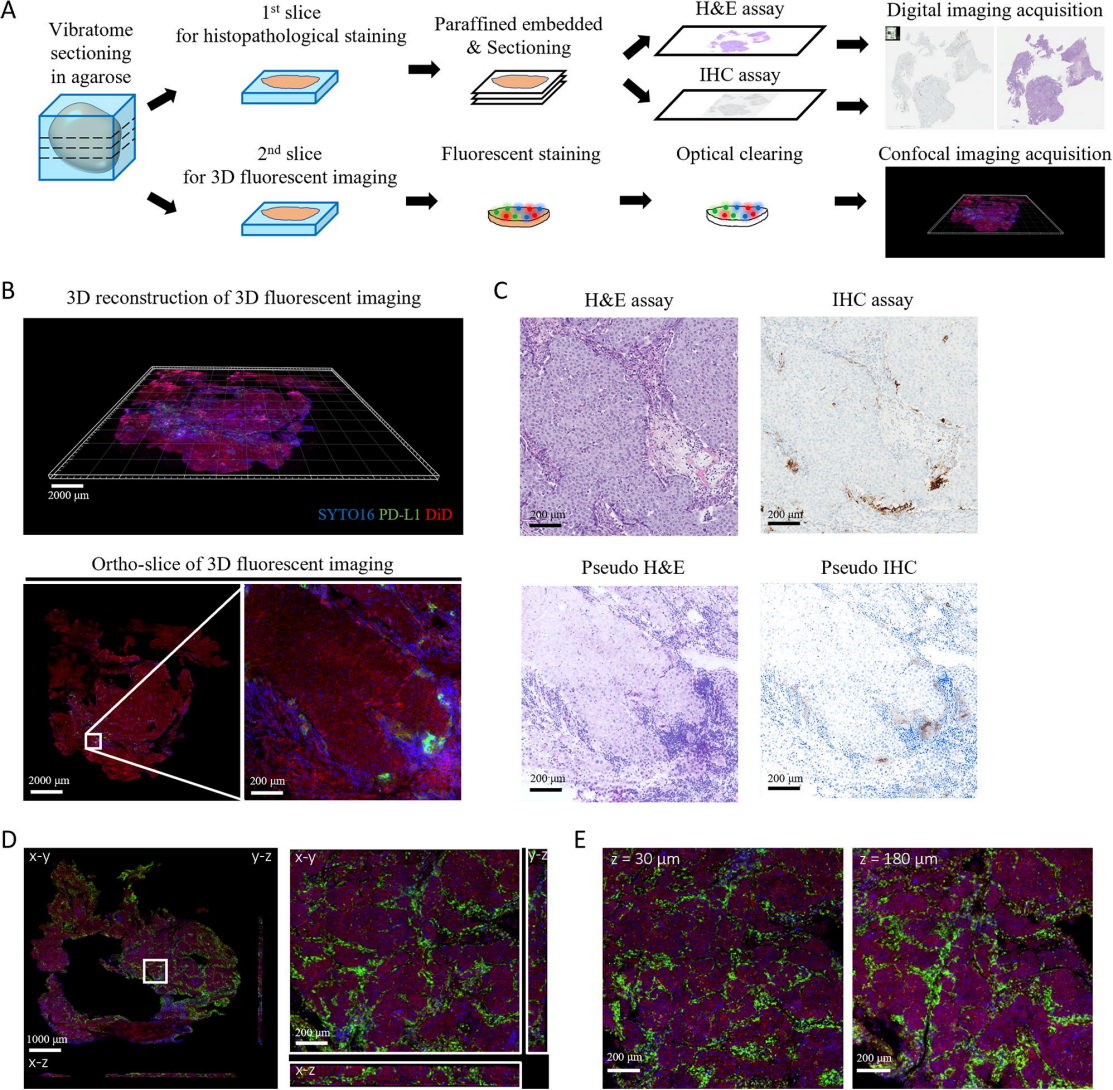
Agreement of the proposed 3D framework with traditional, clinical-grade 2D staining techniques. (**A**) Procedural workflow for conventional 2D analysis (upper row) and 3D imaging of immunofluorescence-stained, optically cleared breast cancer specimens (lower row). After tissue embedding in 3% agarose, a vibratome was used to obtain 200-µm-thick slices. Paired slices (n = 2) from the same patient were subjected to (1) paraffin embedding followed by traditional 2D hematoxylin and eosin (H&E) staining and IHC (n = 1) and (2) 3D immunofluorescence staining (n = 1). PD-L1 is labeled in green color in immunofluorescence images, whereas nuclei and cell membranes were counterstained with SYTO-16 (blue color) and DiD (red color), respectively. (**B**) Illustrative example of immunofluorescence images obtained from case #7 (percentage of tumor-infiltration immune cells = 10%): 3D reconstruction (upper row) and ortho-slice visualization (lower row, left side) showing the region of interest (lower row, right side); scale bar = 2000 μm (upper row and lower row, left side), scale bar = 200 μm (lower row, right side). (**C**) Comparison of real H&E (upper row, left side) and real IHC (upper row, right side) images *versus* pseudo H&E (lower row, left side) and pseudo IHC (lower row, right side) images obtained from the conversion of immunofluorescence images; scale bar = 200 μm. (**D**) Cross-sectional images obtained from case #2 (percentage of tumor-infiltration immune cells = 90%) were characterized by a uniform immunofluorescence staining in the 3D space; scale bar = 1000 μm. (left), scale bar = 200 μm (right). (**E**) Uniform PD-L1 labeling in the top and bottom layers of the sample obtained from case #2; scale bar = 200 μm

Fresh primary tumor specimens were fixed at room temperature (RT) for 12 − 72 h in 10% neutral buffered formalin (Leica biosystems, Richmond, IL, USA) and subsequently placed in phosphate-buffered saline (PBS)-azide buffer (0.02% sodium azide; Sigma-Aldrich, St. Louis, MO, USA) for long-term preservation at 4 °C. After tissue embedding in 3% agarose (Sigma-Aldrich), a vibratome was used to obtain 200-µm-thick slices. Paired slices (n = 2) from the same patient were subjected to (1) paraffin embedding followed by traditional 2D hematoxylin and eosin (H&E) staining and IHC (n = 1) and (2) 3D immunofluorescence staining (n = 1; Fig. 1A).

### Hematoxylin and eosin staining and immunohistochemistry

H&E staining and IHC were performed on 4-µm-thick sections obtained from paraffin-embedded 200-µm-thick slices. For comparison with PD-L1 expression data obtained from 3D immunofluorescence, 2D PD-L1 expression was analyzed using a commercially available kit (PD-L1 [SP142] IHC assay; Ventana Medical Systems, Tucson, AZ, USA) in the same sample subset (n = 20). H&E and IHC sections were digitalized into whole-slide images using an Aperio AT2 slide scanner (Leica Biosystems, Nußloch, Germany). Two experienced pathologists (Lee YH and Huang CY) independently assessed PD-L1 IHC staining.

### 3D immunofluorescence staining and optical clearing

Prior to immunofluorescence staining, all 200-µm-thick slices were exposed to 2% Triton X-100 (J.T. Baker, Radnor, PA, USA) in PBS buffer, and blocked using 3% hydrogen peroxide and 10% goat serum blocking buffer (Thermo Fisher Scientific, Eugene, OR, USA). For tracking PD-L1 expression on tumor-infiltrating immune cells, the slices were initially incubated at 37 ºC for 24 h under continuous shaking (100 rpm) with primary recombinant antibodies (Ventana Medical Systems), followed by exposure to poly-horseradish peroxidase (HRP)-linked goat anti-rabbit/mouse secondary antibodies (IgG; Thermo Fisher Scientific) at RT for 30 h (shaking speed: 100 rpm). The Alexa Fluor™ 555 tyramide reagent (Thermo Fisher Scientific) was used for fluorescence signal amplification. Nuclei and cell membranes were counterstained with SYTO-16 (5 µM; Thermo Fisher Scientific) and DiD (20 µg/mL; Thermo Fisher Scientific) labeling solutions, respectively. For optical tissue clearing, the slices were incubated overnight with a proprietary reagent (JelloX Biotech Inc., Hsinchu, Taiwan) [23] – which was also used to promote tissue adhesion on glass slides prior to image acquisition. We ruled out the occurrence of autofluorescence in the Alexa Fluor™ 555 channel by incorporating negative control specimens (n = 3) that did not undergo primary antibody staining (Fig. S1).

### Standardization of immunofluorescence staining and image acquisition parameters

With the goal of reaching clinical-grade reliability similar to that provided by traditional IHC, a thorough standardization process of the immunofluorescence staining process was implemented to avoid artifacts and signal alterations (Fig. 1A). Staining and image acquisition parameters were initially fine-tuned by analyzing CD45 and PD-L1 expression in ten and six BC specimens, respectively. Following complete optical clearing, the sensitivity and offset were kept fixed during scanning; however, deeper signals required higher laser intensities to achieve excitation. Therefore, slight adjustments of the intensity profile along a fixed slope were performed (Table S1).

### 3D image acquisition

3D image acquisition was performed in the resonant mode (lateral resolution = 0.621 μm) on an FV-3000 confocal laser scanning microscope (Olympus, Tokyo, Japan) equipped with a 20× objective lens. Multiple images were captured at various depths (interval on the z-axis = 1.4 μm). Prior to image data export, stitching and normalization were undertaken using the FV31S-DT (Olympus) and Imaris 9.8 (Bitplane, Belfast, UK) software packages. The pseudo-H&E/IHC images demonstrated in Fig. 1C were transformed from immunofluorescence images by the open-source computer software MetaLite® (JelloX Biotech Inc., Hsinchu, Taiwan).

### Tumor-infiltrating immune cell detection algorithm

**Fig. 2:**
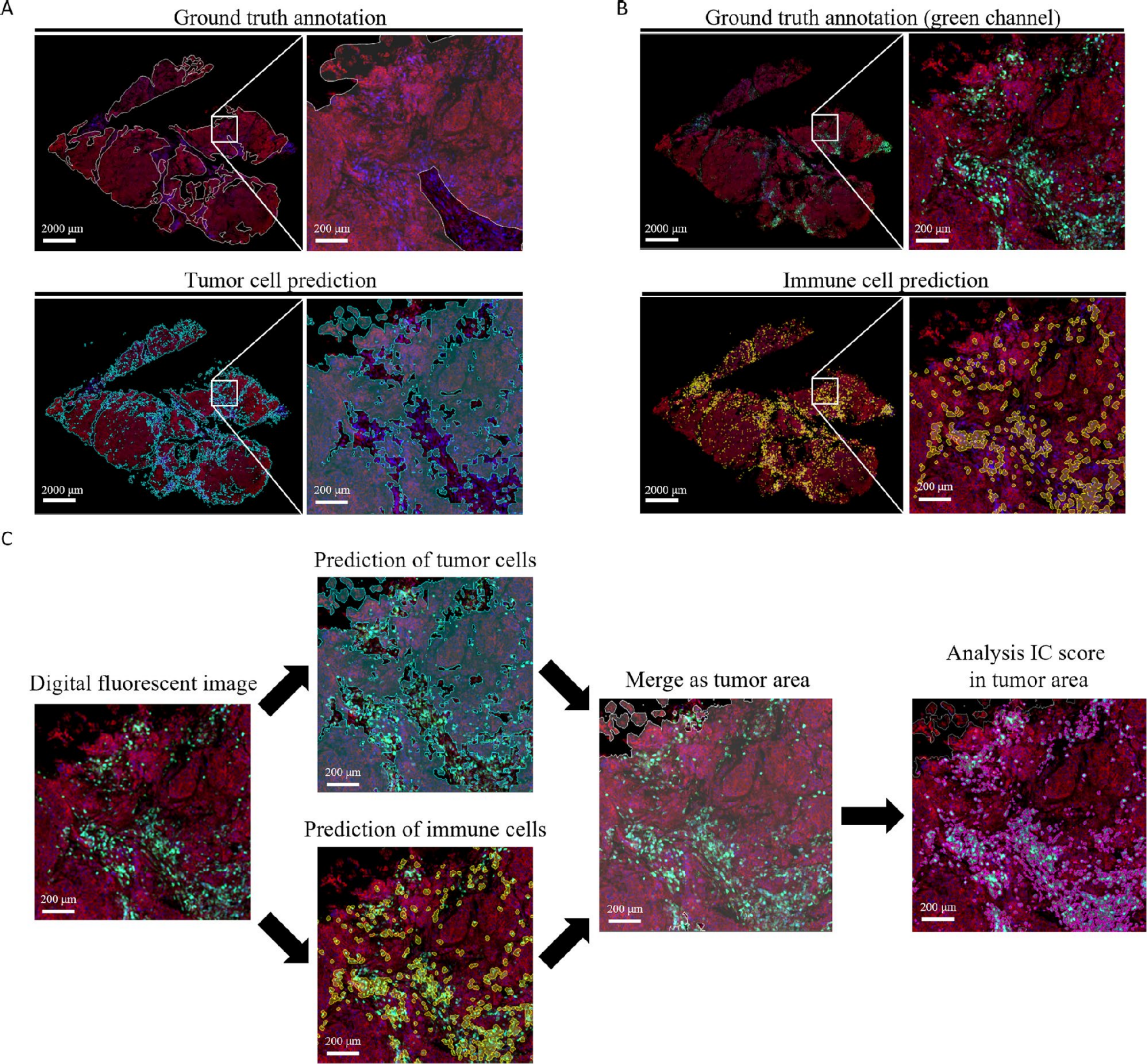
Computer-assisted algorithm for the detection of tumor-infiltrating immune cells in immunofluorescence images. (**A**) The ground truth annotation mask (white) and the tumor cell segmentation model mask (cyan) were visually compared for the detection of tumor areas. (**B**) The ground truth annotation mask (CD45, green fluorescence) and the immune cell segmentation model mask (yellow mask) were visually compared for the detection of tumor-infiltrating immune cells. (**C**) Workflow of the computer-assisted algorithm for the detection of PD-L1 expression. Cyan: tumor segmentation model mask; yellow: immune cell segmentation model mask; white: merged tumor area mask; magenta: CD45-positive tumor-infiltrating immune cells mask. Nuclei and cell membranes were counterstained with SYTO-16 (blue color) and DiD (red color), respectively. Scale bar = 2000 μm (whole view), scale bar = 200 μm (region of interest)

Two machine-learning models (i.e., a tumor cell segmentation model followed by an immune cell segmentation model) were integrated to devise a computer-assisted algorithm for the detection of tumor-infiltrating immune cells in 3D immunofluorescence images. Following SYTO-16 and DiD counterstaining, the algorithm was able to identify tumor regions and immune cells in a stepwise fashion. Subsequently, relying on the Ventana PD-L1 (SP142) assay interpretation guide [24], the algorithm computed the percentage of tumor-infiltrating immune cells by dividing the area of immune cells expressing PD-L1 by the total tumor area. The tumor and immune cell segmentation models were trained using 40 and 38 fluorescent images obtained from 18 to 10 BC specimens, respectively. Ground-truth annotation of tumor regions was performed by three experienced scientists at JelloX Biotech Inc. followed by an independent review by a board-certified pathologist (Lee YH). Expression of CD45 by immunofluorescence staining was used for ground-truth annotation of immune cells. All images were cropped into square patches (size: 256 × 256 pixels) and randomly assigned to different sets for training, validation, and testing in an 8:1:1 ratio (Table S2A). The trained tumor-infiltrating immune cell detection algorithm was subsequently applied to images captured at different depths (interval on the z-axis = 7 μm). Figure 2 summarizes the workflow used to devise the algorithm; the detailed procedure is described in Supplementary Methods and Table S2D.

### Definition of positive PD-L1 expression

PD-L1-expressing tumor-infiltrating immune cells covering ≥ 1% tumor area of invasive ductal carcinoma (IDC) were considered positive for PD-L1 expression, independent of the staining intensity. Samples stained within the > 0–1% interval were considered as borderline, whereas those with a percentage of 0% as negative. The 1% threshold was selected for its clinical significance in the selection of candidates for ICIs. Scoring was performed according to the VENTANA PD-L1 (SP142) assay interpretation guide.

### Outcome measures

The outcome measures for this study included the concordance of the devised methodologies (i.e., 3D immunofluorescence staining and computer-assisted assessment tool) with the traditional 2D techniques (H&E and IHC), assessed using overall percentage agreement, with respect to (1) morphological characteristics ( RGB-colored digital image of immunofluorescence staining *versus* digital image of traditional techniques), (2) detection and phenotypic characterization of immune cells (3D immunofluorescence staining and computer-assisted assessment tool *versus* traditional techniques), and (3) assessment of PD-L1 expression (3D immunofluorescence staining and computer-assisted assessment tool *versus* traditional techniques). Finally, the inter-layer heterogeneity of PD-L1 expression was analyzed in the 3D space.

## Results

### Concordance between 3D immunofluorescence staining and traditional staining techniques

After standardization, 3D immunofluorescence images were assessed by two independent pathologists (Table S3) and found to be visually similar to those obtained with the traditional 2D staining protocols (H&E and IHC; Fig. 1B and C). Among multiple layers of 3D fluorescent images, the most superficial one (adjacent to H&E/IHC sections) was selected for comparison purposes. In the vast majority of cases (95%, 19/20), the morphological characteristics of immunofluorescence-stained samples – as reflected by the presence of ductal carcinoma in situ (DCIS) or IDC – were consistent with those observed in H&E-stained specimens. A high concordance (80%, 16/20) between H&E/IHC and adjacent fluorescent images was also found for classifying PD-L1 expression as either positive or negative (Table S3). Collectively, these results indicated that PD-L1 expression patterns within the tumor immune microenvironment appeared to be consistent across different methodologies. In addition, the fluorescent images showed a high inter-observer reliability, with the two pathologists (Lee YH and Huang CY) classifying PD-L1 expression as identical in all cases (100%, 20/20; Table S3).

When tumor-infiltrating immune cells with PD-L1 expression were abundantly present, a uniform immunofluorescence staining was observed in different layers (Fig. 1D and E). PD-L1 had a prominent cell surface expression detectable at various depths, confirming the consistency of both fluorescent staining and image acquisition.

### Concordance between computer-assisted immune cell detection algorithm and traditional staining techniques

We next examined the ability of the computer-assisted algorithm to detect tumor-infiltrating immune cells in 3D immunofluorescence images (Supplementary Methods). The initial tumor cell segmentation model successfully generated a tumor cell mask (Fig. 2A) with a > 80% accuracy (Table S2B and S2E) for the detection of tumor areas. Thereafter, the immune cell segmentation model achieved 90% classification accuracy (Fig. 2B and Table S2C) for identifying immune cells. The subsequent use of tumor cell and immune cell masks allowed detecting immune cells within the tumor area (i.e., cells identified by the immune cell mask within the area delineated by the tumor cell mask) as well as in the adjacent peritumoral stroma (i.e., cells identified by the immune cell mask outside the area delineated by the tumor cell mask). The percentages of tumor areas occupied by PD-L1-positive cells, calculated by the algorithm for each layer (Fig. 2C), were compared with the results of IHC. On analyzing PD-L1 expression levels on immune cells according to three different categories (≥ 1%, > 0–1%, and 0%), we found a concordance rate of 90% (18/20) between the prediction algorithm and traditional IHC (Table S4). In one of the two misclassified cases (case #13), there was an underestimation of PD-L1 expression level due to the presence of a large DCIS area included by the tumor cell segmentation model.

### Heterogeneous PD-L1 expression patterns in 3D immunofluorescence images

**Fig. 3:**
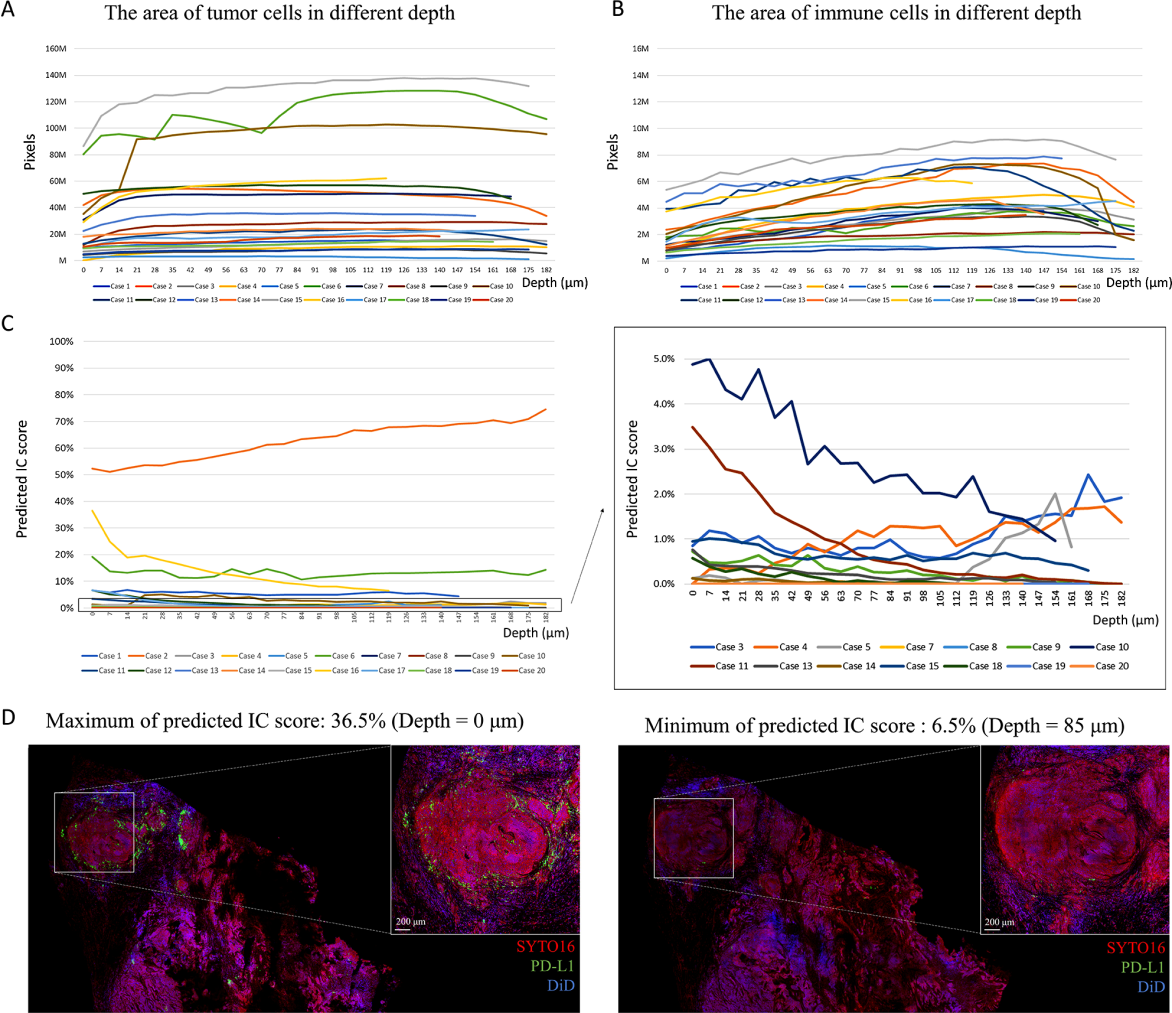
Variation in the predicted immune cell score at different depths of the 3D space. (**A**) Variation of tumor area calculated by the computer-assisted algorithm at different depths of the 3D space. (**B**) Variation of immune cells calculated by the computer-assisted algorithm at different depths of the 3D space. (**C**) Variation in the PD-L1 immune cell score calculated by the computer-assisted algorithm at different depths of the 3D space. (**D**) Maximum observed difference in the immune cell score for different layers (case #16); scale bar = 1000 μm (whole specimen image), scale bar = 200 μm (region of interest image)

With the detection of the computer-assisted algorithm, the areas of tumor cells showed uniformity in each case (Fig. 3A). The immune cell density of most cases was slightly different between each consecutive plane in 3D space, as shown by the smooth curve of quantified areas of immune cells in different depths (Fig. 3B). However, the difference between the maximal and minimal area of immune cells may be significant (> 10%) in a given case when we increase the depth examined. Figure 3C depicts the variation in the immune cell score, as predicted by the algorithm, at different depths of the 3D space. The maximum difference in the immune cell score between different layers was observed in case #16 (Fig. 3D).

**Fig. 4:**
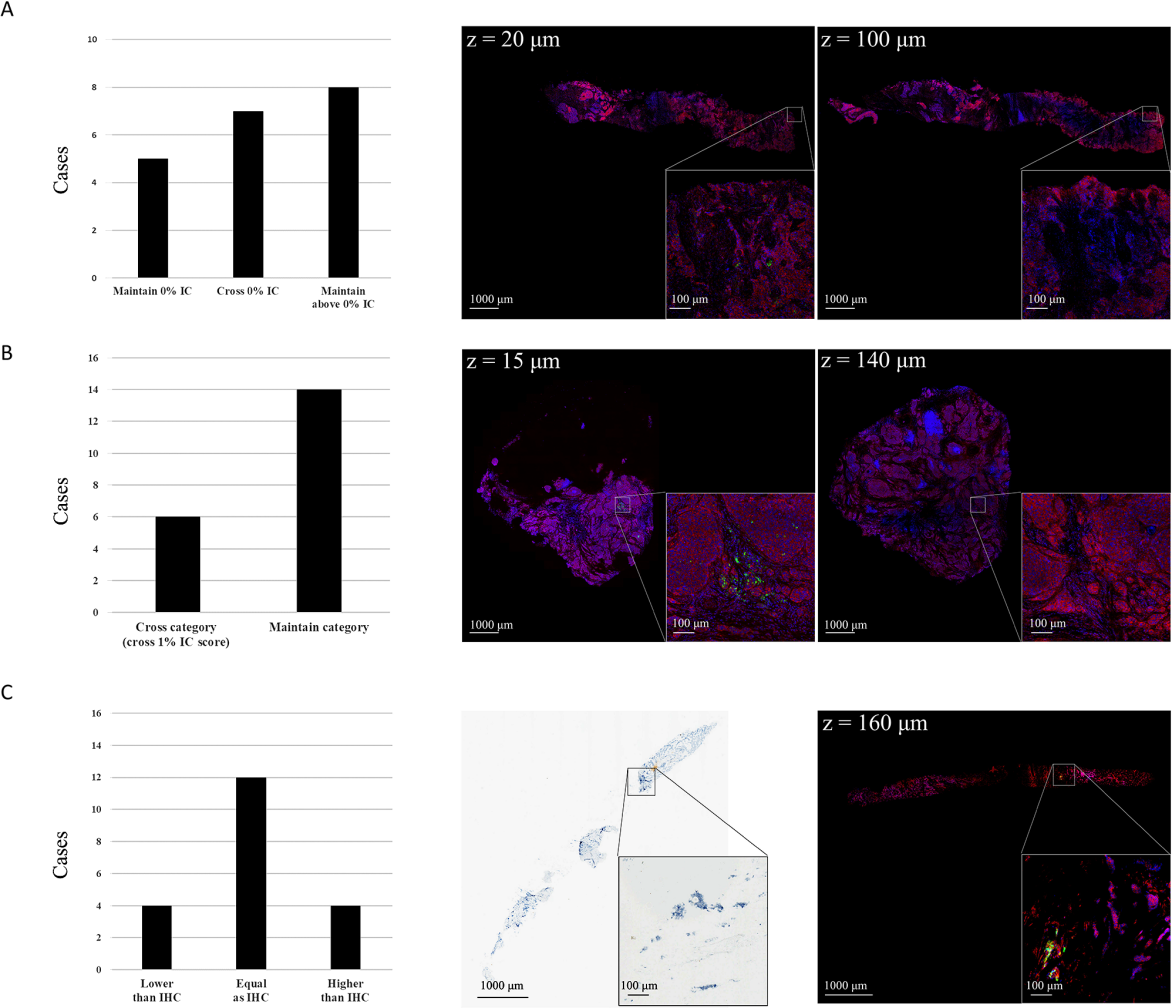
Heterogeneous PD-L1 expression patterns in the 3D space. (**A**) In 7 of 20 cases (35%), PD-L1 SP142 at different depth levels illustrated absence immunofluroescence change to signal expression (left). Case #18 showed no PD-L1 expression at a 20-µm depth (middle) and change into positive PD-L1 expression at a 100-µm depth (right). (**B**) In 6 of 20 cases (30%), PD-L1 SP142 at different depth levels illustrated PD-L1 category (cross 1%) change. Case #17 showed above 1% PD-L1 expression at a 15-µm depth (middle) and change into negative expression at a 140-µm depth (right). (**C**) Eight of 20 cases (40%) illustrated immune cell scores for all levels of 3D fluorescence images were difference from original IHC. Case #5 showed the different PD-L1 expression level in IHC and 3D fluorescent image. Scalebar, 1000-µm (whole specimen image), scale bar = 100-µm (region of interest image)

On analyzing 3D immunofluorescence images, eight of the 20 examined cases (40%) showed a heterogeneous PD-L1 expression pattern. Among which, seven had some layers wherein PD-L1 was expressed (expression levels ≥ 1% or > 0–1%) along with others in which PD-L1 was undetectable. Figure 4A illustrates the most striking example of a heterogeneously expressed PD-L1 (case #18). A consistent lack of expression across all layers was found in five cases only (25%). Notably, there were six cases (30%) wherein PD-L1 expression levels along different layers crossed the 1% threshold for admitting patients to ICIs (Fig. 4B).

### Discrepancy between average PD-L1 expression in 3D immunofluorescence images and immunohistochemistry

For each case, we subsequently calculated the average PD-L1 immune cell score predicted by the algorithm for all layers of the 3D fluorescence image (Table S5). 3D immunofluorescence images and IHC showed discrepant results with respect to PD-L1 expression in 8 of the 20 cases (40%). PD-L1 expression was found to be higher in 3D immunofluorescence images in half of such cases, whereas the opposite was evident for the remaining four (Fig. 4C).

### Subgroup analysis of triple-negative breast cancer

Of the six cases with triple-negative BC included in the study, three were identified by the algorithm as showing inter-layer heterogeneity in terms of PD-L1 expression – with certain layers crossing the 1% threshold. Notably, the algorithm classified as positive one case (Fig. 4B, case #17) originally deemed negative according to IHC. Detailed information for each case is provided in Table S5.

## Discussion

In this proof-of-concept study, we describe a novel computer-assisted algorithm for the 3D assessment of PD-L1 expression in BC specimens using immunofluorescence staining and optical clearing methodologies, and report three principal findings. First, the proposed framework was feasible and showed a high overall agreement with traditional, clinical-grade 2D staining techniques. Second, the results obtained for automated immune cell detection and analysis of PD-L1 expression were satisfactory. Third, the spatial distribution of PD-L1 expression was heterogeneous across various BC tissue layers in the 3D space, and the average expression was different from the results of traditional IHC in a significant proportion (40%) of cases.

While the applications of fluorescence microscopy in the field of BC diagnostic pathology remain limited [25], immunofluorescence staining combined with optical clearing has been increasingly used to establish novel 3D tumor marker models for guiding clinical decisions in oncology [19, 26]. We briefly compared the benefits and drawbacks of traditional IHC assessment and the 3D approach in Table 1 [22]. Although the processing time and cost of the 3D approach were higher than IHC assessment, it is still a viable option for most patients compared to other pathological examinations. The 3D approach has the capacity to ensure the integrity and precision of pathological diagnoses, thereby facilitating precision medicine for both pathologists and patients through the effective utilization of medical resources. Furthermore, the specimens utilized in the 3D approach can be repurposed for downstream assays in previous studies [23, 27].

**Table 1:** Traditional IHC vs. 3D approach

	Traditional IHC	3D approach
Processing cost	3D approach costs 10 times more than IHC per case	
Capital equipment cost	The cost is nearly similar in IHC and 3D approach	
Structural/Molecular information	3D approach provides 45 times more information than a single-section IHC	
Processing time	3 working days	7 working days
Histological information	Fragmentary	Continuous
Image pre-process	Required	Not required
Diagnostic stratification	Less accurate	More accurate
Utilization for downstream assays	Not applicable since destructive	Applicable since non-destructive

However, due to substantial differences between 2D and 3D techniques with respect to sample preparation and imaging approaches, direct comparisons are necessary to ensure that reliable theranostic information can be captured. In addition, overcoming potential artifacts arising from fluorescence cross-talk requires standardization of different technical parameters (e.g., staining time, dye concentration, laser intensity, and contrast regulation) [28, 29]. In a previous report focusing on non-small cell lung cancer [23], we described a novel technology for quantifying PD-L1 expression and tumor proportion score in 3D space. In the current study, the procedural workflow has been further refined to avoid misclassification through the standardization of different technical phases (i.e., fixation, staining, imaging, and image export). Compared with traditional 2D techniques (H&E and IHC) applied to different sections, immunofluorescence imaging of optically cleared sections allows increasing coverage of the specimen and producing high-resolution 3D images. The analysis of BC specimens using the method devised in our study demonstrated high concordance with traditional 2D techniques, suggesting that pathologists can be provided with accurate biomarker information for potential usage to guide treatment decisions (Table S3). However, future quality control studies examining potential hardware-related effects (e.g., laser diode lifetime, charge-coupled device characteristics) [30] are required before clinical application. With strict regulation of a standardized workflow and modifications to the hardware’s quality control, it will be possible to implement the pipeline of 3D approach in other medical centers that possess similar high-quality 3D imaging capabilities.

In recent years, computer-assisted prediction algorithms have been gaining increasing attention in the field of diagnostic pathology [28, 31]. However, obtaining highly specific signals for each channel is a key prerequisite to devise diagnostically reliable algorithms for use with fluorescence imaging. Herein, the integration of a tumor cell segmentation model with an immune cell segmentation model allowed detecting tumor-infiltrating immune cells in 3D immunofluorescence images in an effective and automated fashion. On analyzing the 3D spatial patterns of PD-L1 expression, the algorithm showed a significant inter-layer heterogeneity which was undetectable within the framework of traditional 2D IHC analysis. Notably, the PD-L1 expression level should only include IDC regions according to the assay interpretation guide. The presence of a large DCIS area – which consists of tumor cells – was included by the tumor cell segmentation model, causing an overestimation of IDC tumor area and leading to an erroneous underestimation of PD-L1 expression level. It points out the need to optimize the computer-assisted algorithm in the future due to the limited training database, as the exclusion of tissue morphology from the database may result in inaccurate predictions of IDC regions, including DCIS areas. This issue may be addressed in future studies by training the tumor cell segmentation model to specifically recognize DCIS areas and exclude them from the calculation. Recognition can be accomplished either by morphological analysis or with the use of myoepithelial markers (e.g., p63, CK5/6, or calponin). Moreover, advanced analyses, such as the variation of tumor growth patterns or the distribution of intra- and extra-tumoral immune infiltrate, can also be considered in the future work of the computer-assisted algorithm [32].

Published data from clinical trials showed that patients with low or negligible PD-L1 tumor expression may still respond to anti-PD-L1 blockade treatments [31]. The heterogeneous expression of PD-L1 in malignant cells [15, 33] may account for this unexpected finding. Here, we provided direct proof that PD-L1 expression varies across different tumor layers with smooth transformation of the distribution of immune cells when 3D structures are thoroughly examined, indicating the approach has the potential to provide more accurate diagnosis of PD-L1 in TNBC. Considering that some, but not all, layers crossed the 1% threshold for identifying patients who may truly benefit from ICIs, further research is necessary to identify the most suitable cutoff value for PD-L1 expression in the 3D context. Additional studies are also required to compare its clinical utility with that of other biomarkers for predicting response to immunotherapy [13, 15, 34].

## Conclusion

In conclusion, this original proof-of-concept study has set the stage for assessing the heterogeneity of PD-L1 expression in immunofluorescence-stained and optically cleared BC specimens. Pending further standardization and optimization, we expect that our technology will become a valuable addition for assessing PD-L1 expression in patients with triple-negative BC. Via a single round of immunofluorescence imaging, our approach may provide a considerable improvement in patient stratification for cancer immunotherapy as compared with standard techniques.

### Electronic supplementary material

Below is the link to the electronic supplementary material.

**Supplementary Material 1**: Supplementary Figure 1. Autofluorescence testing of the fluorescent staining and imaging procedure. PD-L1 is labeled in green color, whereas nuclei and cell membranes were counterstained with SYTO-16 (blue color) and DiD (red color), respectively; scale bar = 1000 μm (whole specimen image), scale bar = 150 μm (region of interest image and channel).

**Supplementary Material 2**: Supplementary Table 1. Experimental conditions for image acquisition and fixed adjustment of data export: standard 3D imaging setup using average values from six breast cancer specimens.

**Supplementary Material 3**: Supplementary Table 2. Dataset distributions and confusion matrices of the tumor cell segmentation model and the immune cell segmentation model. (A) Dataset distribution for each model. (B) Confusion matrix of the tumor cell segmentation model assessed by pixelwise metrics in the testing dataset. (C) Confusion matrix of the immune cell segmentation model evaluated by pixelwise metrics in the testing dataset. (D) Selected parameters for the devised computer-assisted method. (E) Pixelwise evaluation metrics of the tumor cell segmentation model for different images in the testing dataset; only the images that were split into more than 30 testing patches are included.

**Supplementary Material 4**: Supplementary Table 3. Similarities between H&E/IHC and adjacent fluorescent images as well as between the two pathologists on the same fluorescent image. For each multilayer 3D fluorescent image, the most superficial layer adjacent to the HE/IHC sections was selected.

**Supplementary Material 5**: Supplementary Table 4. Quantitative analysis of PD-L1 expression. (A) Confusion matrix of computer-assisted algorithm assessed by PD-L1 expression level in each case. (B) Comparison of computer-assisted prediction algorithm versus traditional pathological diagnosis carried out on the same digital fluorescent image.

**Supplementary Material 6**: Supplementary Table 5. Summary of PD-L1 expression values in each breast cancer case.

**Supplementary Material 7**: Supplementary Methods. Design of the tumor-infiltrating immune cell detection algorithm.

## Data Availability

All data supporting the findings of this study are available from the corresponding author upon reasonable request.

## List of abbreviations

2D: Two-dimensional
3D: Three-dimensional
BC: Breast cancer
DCIS: Ductal carcinoma in situ
ER: Estrogen receptor
H&E: Hematoxylin and eosin
HER2: Human epidermal growth factor receptor 2
HRP: Poly-horseradish peroxidase
ICIs: Immune checkpoint inhibitors
IDC: Invasive ductal carcinoma
IHC: Immunohistochemistry
PBS: Phosphate-buffered saline
PD-1: Programmed death-1
PD-L1: Programmed death-ligand 1
PR: Progesterone receptor
RT: Room temperature
