# Supplementary figures and images for: A novel computer-assisted tool for 3D imaging of programmed death-ligand 1 expression in immunofluorescence-stained and optically cleared breast cancer specimens
Source: BMC Cancer. 2024 Jan 24;24:121. doi: 10.1186/s12885-023-11748-8 (PMC10807239; doi:10.1186/s12885-023-11748-8)

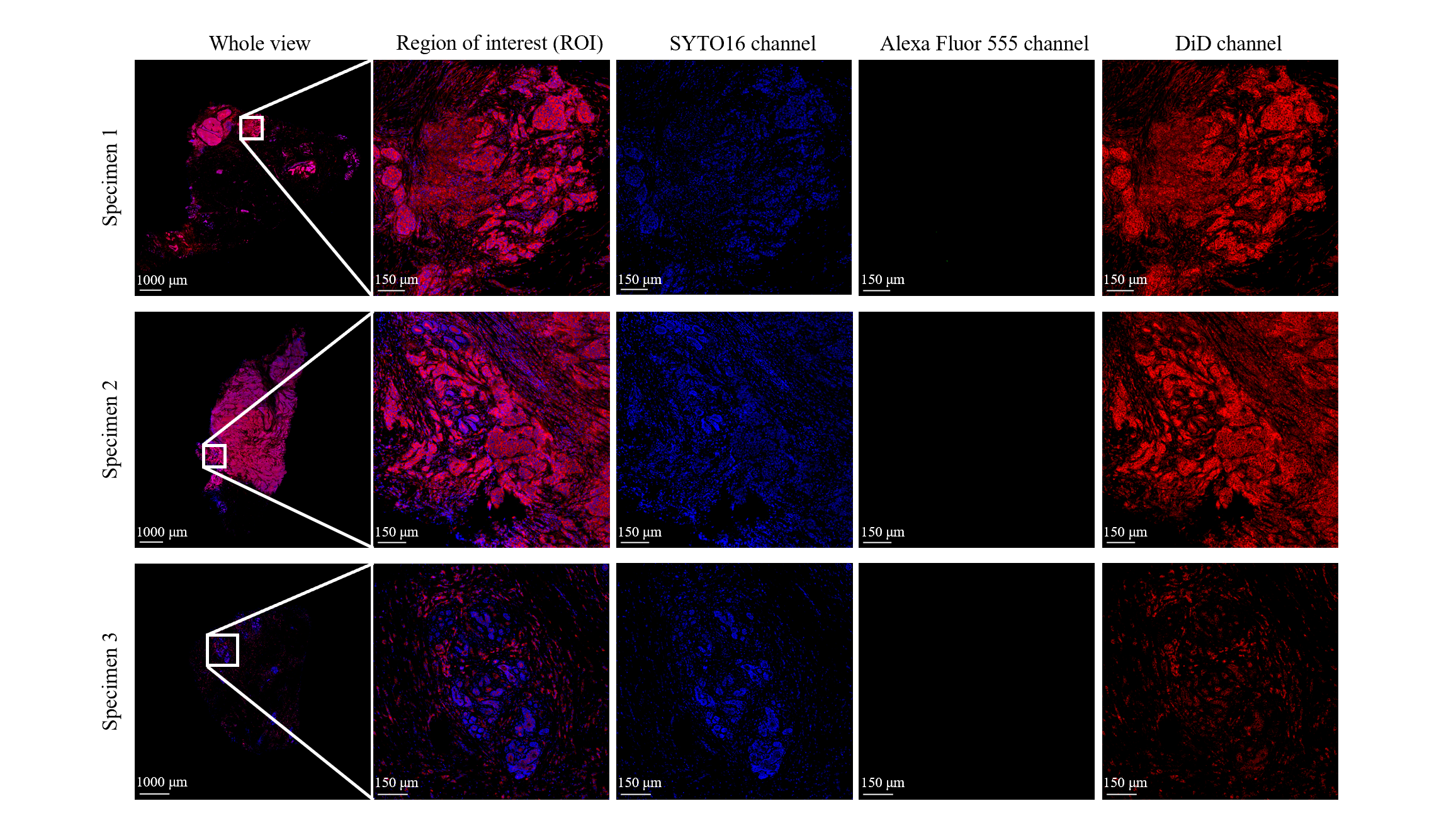

Supplement: Supplementary file 1 — Supplementary Material 1: Supplementary Figure 1. Autofluorescence testing of the fluorescent staining and imaging procedure. PD-L1 is labeled in green color, whereas nuclei and cell membranes were counterstained with SYTO-16 (blue color) and DiD (red color), respectively; scale bar = 1000 μm (whole specimen image), scale bar = 150 μm (region of interest image and channel). [file 12885_2023_11748_MOESM1_ESM.tif]
