# Supplementary material for: A novel computer-assisted tool for 3D imaging of programmed death-ligand 1 expression in immunofluorescence-stained and optically cleared breast cancer specimens
Source: BMC Cancer. 2024 Jan 24;24:121. doi: 10.1186/s12885-023-11748-8 (PMC10807239; doi:10.1186/s12885-023-11748-8)
Supplement: Supplementary file 2 — Supplementary Material 2: Supplementary Table 1. Experimental conditions for image acquisition and fixed adjustment of data export: standard 3D imaging setup using average values from six breast cancer specimens. [file 12885_2023_11748_MOESM2_ESM.pdf]

**Conditions for the Alexa Fluor 555 channel**	
<u>Experimental conditions of imaging acquisition</u>	
Sensitivity (HV)	370
Gain	0
Offset	1
Experimental conditions of laser intensity (%)	Intensity in the upper layer (depth = 15 μm)<br>= 8.0 - 0.0066 × (total thickness/2 - 15 μm)<br>Intensity in the middle layer (total thickness /2) = 8.0<br>Intensity in the lower layer (latest depth = 15 μm)<br>= 8.0 + 0.0166 × (total thickness/2 + 15 μm)
**Conditions for the DiD channel**	
<u>Experimental conditions of imaging acquisition</u>	
Sensitivity (HV)	370
Gain	0
Offset	1
Experimental conditions of laser intensity (%)	Intensity in the upper layer (depth = 15 μm)<br>= 0.4 - 0.005 × (total thickness/2 - 15 μm)<br>Intensity in the middle layer (total thickness /2) = 0.4<br>Intensity in the lower layer (latest depth = 15 μm)<br>= 0.4 + 0.0266 × (total thickness/2 + 15 μm)
**Conditions for the SYTO16 channel**	
<u>Experimental conditions of imaging acquisition</u>	
Sensitivity (HV)	370
Gain	0
Offset	1
Experimental conditions of laser intensity (%)	Intensity in the upper layer (depth = 15 μm)<br>= 0.8 - 0.0083 × (total thickness - 15 μm)<br>Intensity in the middle layer (total thickness /2) = 0.8<br>Intensity in the lower layer (latest depth =15 μm)<br>= 0.8 + 0.0333 × (total thickness + 15 μm)

*Laser intensity changed according to depth: the modification followed a fixed slope between the upper and the middle layers of the middle and lower layer; adjustments were performed using the Bright Z mode of the FV-3000 software.

*Unit of measure for thickness = μm
