# Supplementary material for: A novel computer-assisted tool for 3D imaging of programmed death-ligand 1 expression in immunofluorescence-stained and optically cleared breast cancer specimens
Source: BMC Cancer. 2024 Jan 24;24:121. doi: 10.1186/s12885-023-11748-8 (PMC10807239; doi:10.1186/s12885-023-11748-8)
Supplement: Supplementary file 3 — Supplementary Material 3: Supplementary Table 2. Dataset distributions and confusion matrices of the tumor cell segmentation model and the immune cell segmentation model. (A) Dataset distribution for each model. (B) Confusion matrix of the tumor cell segmentation model assessed by pixelwise metrics in the testing dataset. (C) Confusion matrix of the immune cell segmentation model evaluated by pixelwise metrics in the testing dataset. (D) Selected parameters for the devised computer-assisted method. (E) Pixelwise evaluation metrics of the tumor cell segmentation model for different images in the testing dataset; only the images that were split into more than 30 testing patches are included. [file 12885_2023_11748_MOESM3_ESM.pdf]

(A)

| | Tumor cell segmentation model | Immune cell segmentation model |
|---|---|---|
| Total cases | **18** with PD-L1 staining | **10** with CD45 staining |
| Total images | **40** | **38** |
| Total patches | **52,239** | **67,491** |
| Training dataset | **41,791** patches | **53,993** patches |
| Validation dataset | **5,224** patches | **6,749** patches |
| Testing dataset | **5,224** patches | **6,749** patches |

(B)

| | | Model predictions | | |
|---|---|---|---|---|
| | | Positive | Negative | |
| Label | Positive | **34,450,252** | **10,320,260** | **Sensitivity** 76.95% |
| | Negative | **3,228,232** | **37,591,252** | **Specificity** 92.09% |
| | | | | **Accuracy** 83.84% |

The tumor cell segmentation showed an overall Dice score of 83.57%.

(C)

| | | Model predictions | | |
|---|---|---|---|---|
| | | Positive | Negative | |
| Label | Positive | **9,562,615** | **2516085** | **Sensitivity** 95.95% |
| | Negative | **8,425,145** | **129302952** | **Specificity** 91.63% |
| | | | | **Accuracy** 92.02% |

The immune cell segmentation showed an overall Dice score of 68.42%.

(D)

| Preparation of learning targets for the immune cell group | |
|---|---|
| Kernel size of blurring (SYTO16) | **1** |
| Kernel size of opening (SYTO16) | **5** |
| Kernel size of closing (SYTO16) | **3** |
| Kernel size of blurring (CD45) | **5** |
| Kernel size of opening (CD45) | **5** |
| Kernel size of closing (CD45) | **1** |
| Distance parameter | **5** |
| Overlap proportion parameter | **0.6** |
| **Post-processing of masks predicted by the segmentation models** | |
| Upscaling ratio (tumor) | **2** |
| Iterations of median blurring (tumor) | **2** |
| Downscaling ratio (tumor) | **2** |
| Component size filtering (tumor) | **12800** |
| Upscaling ratio (immune cells) | **8** |
| Iterations of median blurring (immune cells) | **4** |
| Downscaling ratio (immune cells) | **8** |
| Component size filtering (immune cells) | **100** |
| **Approximation of tumor area** | |
| Kernel size of dilation (immune cells) | **21** |
| Distance parameter | **1** |
| Overlap proportion parameter | **0.2** |
| **Approximation of PD-L1 expression area** | |
| Kernel size of blurring (SYTO16) | **1** |
| Kernel size of opening (SYTO16) | **5** |
| Kernel size of closing (SYTO16) | **3** |
| Kernel size of blurring (SP142) | **5** |
| Kernel size of opening (SP142) | **1** |
| Kernel size of closing (SP142) | **5** |
| Component size filtering (SP142) | **256** |
| Distance parameter | **1** |
| Overlap proportion parameter | **0.2** |

(E)

| | **Accuracy** | **Sensitivity** | **Specificity** | **Dice score** |
|---|---|---|---|---|
| Image 1 | 94.71% | 74.27% | 97.09% | 74.54% |
| Image 2 | 92.15% | 91.65% | 92.30% | 84.66% |
| Image 3 | 90.51% | 73.82% | 96.81% | 80.27% |
| Image 4 | 72.03% | 61.20% | 97.14% | 75.36% |
| Image 5 | 77.44% | 79.39% | 72.62% | 83.37% |
| Image 6 | 88.96% | 44.08% | 94.84% | 48.07% |
| Image 7 | 89.14% | 61.28% | 97.29% | 71.86% |
| Image 8 | 94.23% | 88.34% | 95.37% | 83.30% |
| Image 9 | 77.53% | 90.91% | 71.96% | 70.41% |
| Image 10 | 81.54% | 91.60% | 65.81% | 85.83% |
| Image 11 | 89.02% | 92.02% | 87.33% | 85.80% |
| Image 12 | 91.52% | 86.18% | 93.98% | 86.50% |
| Image 13 | 90.41% | 82.34% | 94.98% | 86.13% |
| Image 14 | 89.24% | 68.23% | 96.47% | 75.99% |
| Image 15 | 86.81% | 79.69% | 91.84% | 81.00% |
| Image 16 | 76.88% | 69.99% | 92.02% | 80.55% |
| Image 17 | 79.35% | 77.17% | 90.86% | 86.22% |
| Image 18 | 92.23% | 80.83% | 97.02% | 86.02% |
| Image 19 | 91.37% | 86.30% | 94.75% | 88.90% |
| Image 20 | 67.35% | 53.38% | 97.80% | 69.15% |
| Image 21 | 56.42% | 42.76% | 98.93% | 59.76% |
| Image 22 | 94.05% | 94.70% | 93.29% | 94.51% |
| Image 23 | 93.12% | 95.79% | 87.83% | 94.88% |
| Image 24 | 94.71% | 74.27% | 97.09% | 74.54% |
