# Supplementary material for: A novel computer-assisted tool for 3D imaging of programmed death-ligand 1 expression in immunofluorescence-stained and optically cleared breast cancer specimens
Source: BMC Cancer. 2024 Jan 24;24:121. doi: 10.1186/s12885-023-11748-8 (PMC10807239; doi:10.1186/s12885-023-11748-8)
Supplement: Supplementary file 4 — Supplementary Material 4: Supplementary Table 3. Similarities between H&E/IHC and adjacent fluorescent images as well as between the two pathologists on the same fluorescent image. For each multilayer 3D fluorescent image, the most superficial layer adjacent to the HE/IHC sections was selected. [file 12885_2023_11748_MOESM4_ESM.pdf]

	Similarity between H&E/IHC and adjacent fluorescent images		Similarity between the two pathologists on the same fluorescent image
	Pathologist 1	Pathologist 2	
DCIS Present or not	95%	100%	75%
IDC Present or not	100%	100%	100%
IC of PD-L1 (categorized as <1% or ≥1%)	80%	85%	100%
