# Supplementary material for: A novel computer-assisted tool for 3D imaging of programmed death-ligand 1 expression in immunofluorescence-stained and optically cleared breast cancer specimens
Source: BMC Cancer. 2024 Jan 24;24:121. doi: 10.1186/s12885-023-11748-8 (PMC10807239; doi:10.1186/s12885-023-11748-8)
Supplement: Supplementary file 5 — Supplementary Material 5: Supplementary Table 4. Quantitative analysis of PD-L1 expression. (A) Confusion matrix of computer-assisted algorithm assessed by PD-L1 expression level in each case. (B) Comparison of computer-assisted prediction algorithm versus traditional pathological diagnosis carried out on the same digital fluorescent image. [file 12885_2023_11748_MOESM5_ESM.pdf]

(A)

		Model predictions		
		Positive (≥1%)	Negative (<1%)	
Label	Positive (≥1%)	10 cases	2 cases	**Sensitivity** 83.3%
	Negative (<1%)	0 cases	8 cases	**Specificity** 100%
				**Accuracy** 90.0%

(B)

**Case ID**	**Pathological diagnosis**	**Computer-assisted prediction algorithm**
Case 1	Equal or above 1%	Equal or above 1%
Case 2	Equal or above 1%	Equal or above 1%
Case 3	Equal or above 1%	Equal or above 1%
Case 4	Below 1%	Below 1%
Case 5	Below 1%	Below 1%
Case 6	Equal or above 1%	Equal or above 1%
Case 7	0%	0%
Case 8	0%	0%
Case 9	Below 1%	Below 1%
Case 10	Equal or above 1%	Equal or above 1%
Case 11	Equal or above 1%	Equal or above 1%
Case 12	Equal or above 1%	Equal or above 1%
Case 13	Equal or above 1%	Below 1%
Case 14	Equal or above 1%	Below 1%
Case 15	Equal or above 1%	Equal or above 1%
Case 16	Equal or above 1%	Equal or above 1%
Case 17	Equal or above 1%	Equal or above 1%
Case 18	Below 1%	Below 1%
Case 19	0%	0%
Case 20	0%	0%
