# Supplementary material for: A novel computer-assisted tool for 3D imaging of programmed death-ligand 1 expression in immunofluorescence-stained and optically cleared breast cancer specimens
Source: BMC Cancer. 2024 Jan 24;24:121. doi: 10.1186/s12885-023-11748-8 (PMC10807239; doi:10.1186/s12885-023-11748-8)
Supplement: Supplementary file 7 — Supplementary Material 7: Supplementary Methods. Design of the tumor-infiltrating immune cell detection algorithm. [file 12885_2023_11748_MOESM7_ESM.pdf]

Computer-vision-assisted extraction of fluorescent signals

We applied a computer-vision-assisted approach to extract various signals – including SYTO16, CD45, and SP142 – from fluorescent images. Specifically, the fluorescent signal was processed through (a) median and Gaussian blurring, (b) histogram equalization, (c) binary Otsu thresholding computed over the 3D fluorescent images for SP142 only (36), and (d) opening and closing operations. This procedure generated a mask that calibrated a specific fluorescent signal for each image. A different computer-vision-assisted method was devised to identify the portions of a source fluorescent signal that were attached to another target signal. The processed binary mask of the target signal was initially dilated using a distance parameter. The connected components (i.e., isolated pixel blocks) were subsequently determined in the source binary mask. A source-connected component was deemed attached to the target signal when more than a prespecified portion of its area overlapped with the dilated target mask.

Architecture designs of tumor cell and immune cell segmentation models

The core tumor cell and immune cell segmentation models for the computer-assisted IC algorithm were adapted from the existing Lite-HRNet architecture (37). Lite-HRNet – a deep learning model capable of aggregating information across different image resolutions – is characterized by suitable performance characteristics for medical applications. The Lite-HRNet-18 architecture was combined with the segmentation

head of HRNetV2 (38) – resulting in over 4.7 million learnable parameters for both tumor cell and immune cell segmentation models.

Model training and evaluation

The learning targets of the tumor cell and immune cell segmentation models were prepared separately. Ground-truth tumor cell regions were manually annotated by experienced pathologists, whereas ground-truth annotations of immune cell regions were processed from CD45 immunofluorescence staining. Specifically, the binary masks for the CD45 and SYTO16 signals were generated by applying the proposed computer-vision-based approach. The SYTO16 signal attaching to CD45 was used as the learning target for the immune cell segmentation model. Conversely, the training sample images for both segmentation models only relied on the SYTO16 and DiD fluorescent channels – without information concerning CD45 staining.

Prior to model training, training images and learning targets were divided into small patches. Each patch consisted of 128 × 128 pixels, with neighboring patches overlapping by 20% with respect to width/height (equivalent to 25 pixels). All of the background patches with no fluorescent staining and 90% of patches that included no learning target pixels were discarded. The remaining patches were randomly divided (8:1:1 ratio) into the training, validation, and testing datasets (Supplement 5A). Additionally, training images were augmented on their saturation, brightness, and

contrast; to this aim, each parameter was adjusted by a uniformly sampled factor between 0.7 and 1.3.

The tumor cell and immune cell segmentation models were trained over 100 epochs, and each training step consisted of a 64-patch batch. We used the binary cross entropy loss function as the learning objective for both models; positive and negative pixels were inversely weighted according to their counts in the training dataset. During training, the learning rate was adjusted through a cosine decay schedule with restarts (37). The initial leaning rate was set to 0.002 and decayed to zero within 1300 steps; subsequently, each restart halved the initial learning rate and doubled the decaying period. After training, the model checkpoint with the least validation loss was retained as the segmentation model for the computer-assisted IC algorithm. The hyperparameters of both models were adjusted jointly over their validation patch dataset and 16 fluorescent images from breast cancer specimens, which led to a pixelwise classification threshold of 0.7 and 0.5 for the tumor cell and immune cell segmentation models, respectively. The two trained models were subsequently evaluated through the testing datasets. The model accuracy, specificity, and sensitivity are presented in Supplements 5B and 5C.

Tumor-infiltrating immune cell detection algorithm

The detailed parameters used for devising the IC detection algorithm are shown in Supplement 5D. In brief, the SYTO16 and DiD channels in each fluorescent image were separated from the SP142 channel. The image was subsequently sectioned into patches (size: 128 × 128 pixels) from which the predicted masks of tumor cell and immune cell regions were generated via the segmentation models; to this aim, the tuned classification threshold was applied. Both predicted masks were processed with edge smoothing – which was based on the following operations performed sequentially: upscaling, median blurring, and downscaling. Smoothed masks were filtered to exclude small connected components.

Next, we approximated the tumor area and the area covered by PD-L1-expressing immune cells using computer-vision-based methods. Specifically, the predicted mask of immune cell regions was dilated, and its connected components attached to the predicted tumor regions were determined. The tumor area was defined as the union between the predicted tumor cell regions and attached immune cell regions, whereas the area of PD-L1 expression was calculated by extracting SP142 and SYTO16 signals from the fluorescent image. To this aim, the union of the SP142 binary mask and its attached SYTO16 signal located outside of the tumor cell regions predicted by the segmentation model was taken into account. Finally, the predicted IC score was calculated by dividing the area of PD-L1 expression by the tumor area.
